# Estimating Fitness by Competition Assays between Drug Susceptible and Resistant *Mycobacterium tuberculosis* of Predominant Lineages in Mumbai, India

**DOI:** 10.1371/journal.pone.0033507

**Published:** 2012-03-27

**Authors:** Purva Bhatter, Anirvan Chatterjee, Desiree D'souza, Monica Tolani, Nerges Mistry

**Affiliations:** The Foundation for Medical Research, Mumbai, Maharashtra, India; Queen Mary University of London, United Kingdom

## Abstract

**Background:**

Multi Drug Resistant Tuberculosis (MDR TB) is a threat to global tuberculosis control. A significant fitness cost has been associated with DR strains from specific lineages. Evaluation of the influence of the competing drug susceptible strains on fitness of drug resistant strains may have an important bearing on understanding the spread of MDR TB. The aim of this study was to evaluate the fitness of MDR TB strains, from a TB endemic region of western India: Mumbai, belonging to 3 predominant lineages namely CAS, Beijing and MANU in the presence of drug susceptible strains from the same lineages.

**Methodology:**

Drug susceptible strains from a single lineage were mixed with drug resistant strain, bearing particular non synonymous mutation (*rpoB* D516V; *inhA*, A16G; *katG*, S315T1/T2) from the same or different lineages. Fitness of *M.tuberculosis (M.tb)* strains was evaluated using the difference in growth rates obtained by using the CFU assay system.

**Conclusion/Significance:**

While MANU were most fit amongst the drug susceptible strains of the 3 lineages, only Beijing MDR strains were found to grow in the presence of any of the competing drug susceptible strains. A disproportionate increase in Beijing MDR could be an alarm for an impending epidemic in this locale. In addition to particular non synonymous substitutions, the competing strains in an environment may impact the fitness of circulating drug resistant strains.

## Introduction

Fitness, virulence and infectiousness of a pathogen are defined as its ability to cause and transmit a disease. A strain's fitness is also a reflection of the robustness of its biological system that continues to function, survive and/or reproduce despite accumulating mutations, environmental change and internal noise [1]. This robustness/fitness in *M.tb* has been assessed and translated using in-vitro competition assays measuring the ‘fitness’ of the strains.

There are several factors that are known to affect the fitness of circulating strains: drug pressures, environmental changes, genotype of the strain and the stress induced by the competing strains. All these factors either independently or together put the bacterial population under fluctuating selection pressure [2]. Strain diversity and mutations ensure that some bacteria survive the adverse conditions and become the predominant population. Such survivors tend to have a greater fitness index and thus a greater ability to transmit the disease [3]. These survivors also pose a threat to disease control as the common measures to tackle the disease do not hold true for them.

An example of greater fitness is the efficacy of Beijing strains which acquire drug resistance, evade BCG vaccination and also disseminate more efficiently [4]–[5]. Additionally drug resistant *M. tb* displays diverse fitness, wherein some strains were more fit than the drug susceptible wild type strains [6]. The enhanced fitness of these circulating drug resistant strains in a community could give rise to MDR TB epidemics [7]. This mechanism of enhanced fitness of drug resistant strains has been explained earlier [6], [8]

All studies conducted till date have been focused either on standard laboratory generated mutant strains or particular lineages such as the W/Beijing [9] due to its association with disease severity, MDR and large epidemic outbreaks. There is currently a lack of information on the relative fitness of strains originating from TB endemic areas such as metropolitan Mumbai. Fitness of strains may be affected by high levels of drug resistance [10]–[12], strain heterogeneity along with a significant level of clustering [13]–[15] and host variability. Fitness studies in endemic regions will not only help in predicting the success of tuberculosis control programs but also in predicting ‘strain replacement’ and thus the efficacy of new vaccine candidates [16]. All these factors along with other environmental factors allow several strain types to co-exist but only a select few to predominate.

There are several assays to estimate fitness of strains. However it is debatable as to which assay best simulates *in vivo* conditions. In competition experiments, it is usually assumed that the competing strains do not affect each other and that they compete only by their intrinsic growth rate and efficiency in utilizing available nutrients. Thus we have chosen growth rate as a measure of relative fitness for competing strains *in vitro* [17].

The aim of the present study therefore was to measure and compare growth of drug resistant and susceptible *M.tb* strains of predominant genotypes {MANU, CAS & Beijing (23, 9 & 4% of total strains respectively)} in the TB endemic region of Mumbai in Western India, and to determine whether the genotype of the competing drug susceptible strain has an impact on the fitness of drug resistant strains from the same or different genotypes. It may be recapitulated that MANU was the predominant lineage and Beijing was significantly associated with MDR [14].

## Materials and Methods

### Ethics Statement

Clearance for this study was obtained from the Foundation for Medical Research (FMR) Institutional Ethics Committee (20.07.2001/01).

### Study population

A total of 723 isolates were collected from patients reporting to RNTCP (Revised National Tuberculosis Control program) health posts in 4 municipal wards of Mumbai. Of these 24% of previously untreated, new cases and 41% of first time treatment failures (new cases that have remained sputum positive at the end of anti TB therapy) were reportedly diagnosed with MDR TB [10]. The population thus represents an endemic region with high burden of MDR TB.

### Strain Selection

Strains isolated from new TB patients (n = 646) were subjected to molecular fingerprinting through spoligotyping and Drug Susceptibility Testing (DST) by the BACTEC MGIT 960 method for the four first line anti tuberculosis drugs viz, Isoniazid (H), Rifampicin (R), Ethambutol (E) and Pyrazinamide (Z) [10]. Spoligotyping identified 4 major clusters in the region, which were identified using Spoldb4 as the following lineages namely: MANU (23%), CAS (9%), Beijing (4%), and EAI-5 (4%) [14]. Of these CAS, Beijing and MANU strains resistant to HR+E/Z and 2/3, drug susceptible isolates from each of the 3 lineages with particular non synonymous substitutions in the *rpoB, inhA and katG* locus were chosen for the intra and inter lineage strain competition assays. The laboratory standard strain H37Rv was used as a drug susceptible control.

### Genotypic Study

To ensure that mutations in *rpoB*, *inhA*, and *katG* genes did not influence the fitness of drug resistant strains, 3 drug resistant strains were chosen with identical mutations in these regions. These were the most frequently observed mutations in these loci which were identified using a commercial line probe assay (GenoType MTBDR plus, Hain Life sciences) [18]. The details of the same have been presented in Table 1.

**Table 1:** Characteristics of 12 strains from endemic region of Western India: Mumbai.

Strain ID	Resistance to	Cluster- Spoligotyping	Mutation in *rpo β* gene	Mutation in *kat G* gene	Mutation in *inh A* gene
H37Rv	Susceptible	-	Wild type	Wild type	Wild type
Cs1	Susceptible	CAS	Wild type	Wild type	Wild type
Cs2					
Cs3					
C-MDR	HERZ		D516V	S315T1	A16G
				S315T2	
Bs1	Susceptible	Beijing	Wild type	Wild type	Wild type
Bs2					
B-MDR	HERZ		D516V	S315T1	A16G
				S315T2	
Ms1	Susceptible	MANU	Wild type	Wild type	Wild type
Ms2					
Ms3					
M-MDR	HERZ		D516V	S315T1	A16G
				S315T2	

The table describes the strains selected for the study along with the genotypic mutations found using the Genotype MTBDR plus line probe assay.

Cs1, Cs2, Cs3 are CAS drug susceptible strains.

Bs1, Bs2 are Beijing drug susceptible strains.

Ms1, Ms2, Ms3 are MANU drug susceptible strains.

C-MDR, B-MDR, M-MDR are CAS, Beijing and MANU drug resistant strains respectively.

Wild type indicates presence of all the bands on the hybridization membrane.

HERZ stand for Isoniazid, Ethambutol, Rifampicin and Pyrazinamide respectively.

In our study, all the drug resistant and susceptible strains bore the same drug susceptibility profile when compared using Phenotypic (BACTEC MGIT TB 960) and genotypic (Genotype MTBDR plus, Hains life sciences) methods (data not shown).

### Strain Cultivation and growth

The strains were taken from Lowenstein-Jensen medium (Hi-Media, India) and inoculated into Middlebrook 7H9 (MB7H9) (Becton Dickinson, USA) supplemented with ADC (Alanine Dextrose and Catalse) (Becton Dickinson, USA), 0.5% glycerol broth for 3–4 weeks at 37°C. The cultures were maintained on a shaker. The culture suspension was vortexed and the turbidity was measured. The sample was preserved in MB 7H9 broth at an O.D_600_ = 0.6 at −70°C. A 15% glycerol stock was used for cryo-preservation. CFU were counted for the preserved vial. During assays, strains were used from these preserved stocks defrosted and inoculated for further use.

### *In vitro* competition assay

The drug susceptible strains from a single lineage in combination with drug resistant strains from the same or different lineages were used for competition as follows: Ten ml of MB7H9 broth, supplemented with ADC, 0.5% glycerol, were inoculated separately in two 20 ml roller tubes, with an aliquot of rifampicin-susceptible and an aliquot of rifampicin resistant strains such that the cell density in the tube was 10^5^ CFU/ml. These controls independently depicted that the rifampicin susceptible and rifampicin resistant strains were not retarded in growth. Simultaneously, equal amounts (∼0.5×10^5^ CFUs/ml) were co-inoculated into a new 20 ml roller tube containing 10 ml MB7H9 broth supplemented with ADC and 0.5% glycerol. The separately inoculated cultures were serially diluted on days 0, 3, 7, 10 & 14 and plated in triplicate on petri dishes containing 25 ml of Middlebrook 7H11(MB7H11) (BD,USA) medium supplemented with OADC (Oleic Acid Dextrose and Catalase, Becton Dickinson, USA). The mixed cultures were serially diluted and plated in triplicate on petri dishes containing 25 ml of MB7H11medium with and without 1 µg/ml of rifampicin.

### Framework of Analysis

#### I. Calculation of Relative Fitness

For each competition experiment the mean of three CFUs was used for the calculation of relative competitive fitness. At all time points, the CFU counts on rifampicin plates indicated the number of rifampicin resistant cells in the mixed cultures. The number of susceptible cells was calculated by subtracting the number of resistant cells from the total cell numbers revealed by CFU counts of the plain plates (MB7H11 without rifampicin).

The relative competitive fitness W of the drug resistant strains compared to the drug susceptible strain was calculated using the formula [6]

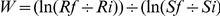

*Ri* and *Si* denote resistant and susceptible cells at baseline (day0) respectively.

*Rf* and *Sf* denote resistant and susceptible cells at endpoint (day14) respectively.

Means of 3 replicate competition assays were determined.

The variance in between the means of samples was analyzed using ANOVA. Difference between the means were analyzed by t-test. The drug resistant strain was considered fit than the drug susceptible strain if its fitness index was more than 1 (the fitness index of drug susceptible strain).

#### II. Calculation of generation time

For both drug susceptible and resistant strains the generation time (g) was calculated using the following formula,
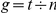
Where, *t* = total time; *n* = no of generations.
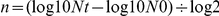

*Nt* = Number of viable cells at final time t; *N0* = Number of viable cells at time 0.

## Results

### Fitness of CAS, Beijing and MANU drug resistant strains

#### In the presence of H37Rv (Figure 1)

**Figure 1:**
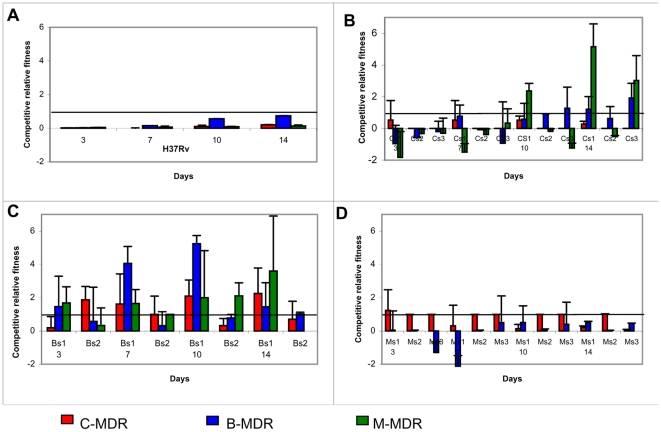
Competitive Relative Fitness (CRF) of drug resistant strains. The relative fitness of drug resistant strains is evaluated in competition with drug susceptible strains (fitness Index 1, represented as a line parallel to X-axis) from the 3 predominant lineages. Cs1, 2, 3 are CAS drug susceptible strains; Bs1, 2 are Beijing drug susceptible strains; Ms1, 2, 3 are MANU drug susceptible strains; C-MDR, B-MDR, M-MDR are CAS, Beijing & MANU drug resistant strains respectively. The values of relative fitness indices have been presented in Table 1.The values are represented as mean ± SD of 3 independent experiments. (Figure 1**A**) represents the fitness index of H37RV in presence of the 3 drug resistant strains. (Figure 1**B**) represents the fitness index of drug resistant strains in the presence of CAS drug susceptible strains. (Figure 1**C**) represents the fitness index of drug resistant strains in the presence of Beijing drug susceptible strains. (Figure 1**D**) represents the fitness index of drug resistant strains in the presence of MANU drug susceptible strains.

In comparison to the standard laboratory strain H37Rv (susceptible to all first line anti-TB drugs, **Table 1**), the drug resistant strains had a reduced fitness. However we found an overall significant difference in the fitness indices of the 3 drug resistant strains with Beijing (B-MDR) being the fittest followed by CAS (C-MDR) and MANU (M-MDR) respectively (ANOVA, F_2,9_ = 4.84, p = 0.03).

#### In the presence of drug susceptible strains from lineages (Figure 1 & Table 2)

**Table 2:** Fitness indices of drug resistant strains in the presence of drug susceptible strains from the 3 predominant strain lineages.

	C-MDR	B-MDR	M-MDR
**H37Rv**	0.2±0.02	0.7±0.003	0.13±0.05
**Cs1**	0.26±0.18	1.21±0.80	5.15±1.44
**Cs2**	0.00±0.00	0.63±0.75	−0.51±0.06
**Cs3**	0.00±0.00	1.92±0.93	3.02±1.57
**Bs1**	2.24±1.53	1.45±1.45	3.60±3.30
**Bs2**	0.70±1.08	1.00±0.10	1.40±1.75
**Ms1**	0.21±0.09	0.50±0.08	0.00±0.00
**Ms2**	0.00±0.03	0.02±0.02	0.00±0.00
**Ms3**	0.08±0.01	0.44±0.03	0.00±0.00

The values represent fitness indices of drug resistant strain in the presence of drug susceptible strains from the 3 predominant lineages. The data is represented as a mean ± SD derived from 3 independent experiments. The values represent fitness index on day 14 and have been used to plot Figure 1.

Cs1, Cs2, Cs3 are CAS drug susceptible strains.

Bs1, Bs2 are Beijing drug susceptible strains.

Ms1, Ms2, Ms3 are MANU drug susceptible strains.

C-MDR, B-MDR, M-MDR are CAS, Beijing and MANU drug resistant strains respectively.

We observed an overall significant difference in the fitness indices of the drug resistant strains (C-MDR, B-MDR, M-MDR) in the presence of drug susceptible strains from 3 different lineages (ANOVA, F_2,6_ = 29.98, p<0.05). The inhibition was prominent in the presence of drug susceptible strains belonging to the same lineage. The growth rate of MANU drug susceptible strain was found to be significantly greater (ANOVA, F_2, 57_ = 3.96, p<0.05) than drug susceptible strains of the CAS and Beijing lineage when in competition with the drug resistant strains of the same and different lineages.

The CAS drug resistant strains demonstrated a reduced fitness index in the presence of drug susceptible strains from all 3 lineages. No drug resistant strains from any of the 3 lineages were inhibited in the presence of Beijing drug susceptible strain. In the presence of the 2 drug susceptible MANU strains (Ms1 &Ms2) the growth inhibition was observed only in the drug resistant M-MDR strain. In contrast the competing combine of the third MANU drug susceptible strain (Ms3) and M-MDR resulted in no growth of either strain.

### Generation time of strains in independent and competition culture (Table 3, 4; Figure 2, 3)

**Figure 2:**
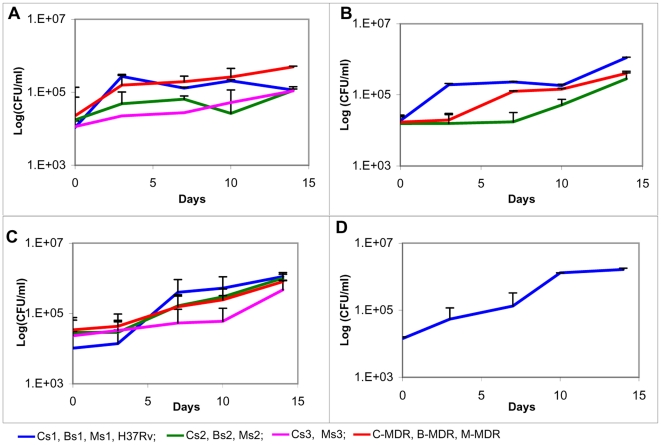
Independent growth curves of drug susceptible and drug resistant strains. The growth curves measured by plating for CFU of the drug resistant and susceptible strains in 7H9 broth over a period of 14 days have been plotted against time. The data is represented as mean ± SD of 3 independent experiments. (Figure 1**A**) represents growth curves of drug susceptible and resistant strains of CAS lineage in single culture. (Figure 1**B**) represents growth curves of drug susceptible and resistant strains of Beijing lineage in single culture. (Figure 1**C**) represents growth curves of drug susceptible and resistant strains of MANU lineage in single culture. (Figure 1**D**) represents growth curve of H37Rv in single culture.

**Figure 3:**
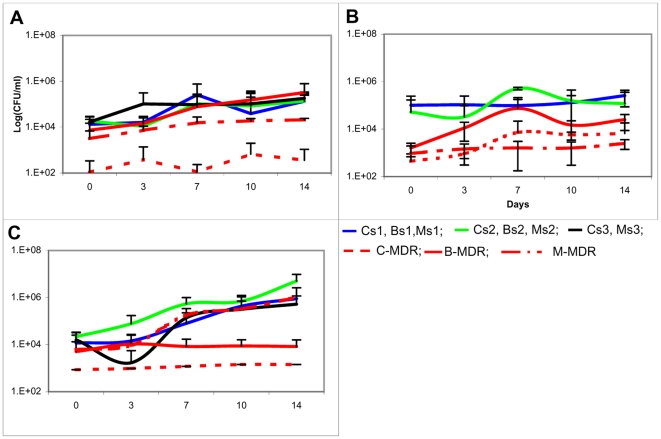
Growth curves of drug susceptible and drug resistant strains in competition. The growth curves of drug resistant and susceptible strains measured by plating for CFU in 7H9 broth in competition over a period of 14 days have been plotted against time. The data is represented as mean ±SD of 3 independent experiments. (Figure 1**A**) represents the growth curves of drug susceptible strain of CAS lineage in competition with the drug resistant strains of the CAS, Beijing and MANU lineage. (Figure 1**B**) represents the growth curves of drug susceptible strain of Beijing lineage in competition with the drug resistant strains of the CAS, Beijing and MANU lineage. (Figure 1**C**) represents the growth curves of drug susceptible strain of MANU lineage in competition with the drug resistant strains of the CAS, Beijing and MANU lineage.

**Table 3:** Mean generation time of independent strains in axenic culture.

Sr no	Strain ID	Generation time (h)Mean ± SD
1	H37Rv	50.8±6.2
2	Cs1	98.7±9.5
3	Cs2	125.5±1.3
4	Cs3	103.5±1.3
5	Bs1	96±6.3
6	Bs2	80.4±3.7
7	Ms1	50±2.9
8	Ms2	65.7±9.3
9	Ms3	77.3±7.7
10	C-MDR	121.2±11.3
11	B-MDR	73.7±6
12	M-MDR	73.9±3.9

The values represent the generation time of all strains independently. The data has been represented as mean ± SD from 3 independent experiments and has been used to plot Figure 2.

Cs1, Cs2, Cs3 are CAS drug susceptible strains;

Bs1, Bs2 are Beijing drug susceptible strains;

Ms1, Ms2, Ms3 are MANU drug susceptible strains;

C-MDR, B-MDR, M-MDR are CAS, Beijing and MANU drug resistant strains respectively.

**Table 4:** Generation time of strains in competition in axenic media.

	Generation Time (h) Mean ± SD			
Susceptible→	*H37Rv*	*CAS*	*Beijing*	*MANU*
Resistant↓				
CAS (C-MDR)	*29.2±2.3*	*82.2±18.7*	*82.2±11.2*	*100.4±15.1*
	**201.02±1.7**	**193.4±11.7**	**70.15±22.3**	**84.5±16.9**
Beijing (B-MDR)	*28.1+0.9*	*91.9+2.7*	*68.2+2.4*	*59.8+13.1*
	**52.5±1.9**	**142.7±9.7**	**87.8±18.7**	**71.4±35.3**
MANU(M-MDR)	*29.8±0.6*	*50.9±23.7*	*72.8±49.9*	*39.7±8.2*
	**373**±**14.7**	**84.7**±**10.6**	**121.8**±**38.5**	**No growth**

The italicized values are the median generation times of the drug susceptible strains in the presence of the respective drug resistant strains. The values formatted as bold are the mean generation times of the drug resistant strain in the presence of the respective drug susceptible strain. The data has been represented as mean ± SD from 3 independent experiments. The values have been used to plot Figure 3.

Generation time is calculated using the formula:
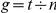

*t* = total time; *n* = no of generations.
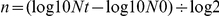

*Nt* = Number of viable cells at final time t; *N0* = Number of viable cells at time 0.

There was a significant difference (p<0.043, t-test) in the generation time of the strains belonging to different lineages. CAS had the longest generation time amongst the 3 lineages. Additionally, the doubling times of C-MDR & M-MDR strains were greatly reduced in the presence of respective drug susceptible strains. Though the difference in doubling times of the strains independently and in competition was not statistically significant (p = 0.427, t-test) there was a uniform trend observed of drug susceptible strains growing faster in competition and drug resistant strains growing faster independently.

## Discussion

Earlier studies have elucidated the putative advantage that drug resistant strains possess due to the presence of certain non-synonymous substitutions in the *rpoB* locus. Elsewhere, reports have highlighted the fitness of drug resistant strains such as the ‘Beijing’ from epidemic regions which are most often single strain outbreaks^9^. However, what remains to be elucidated is the fitness of drug resistant strains in the presence of drug susceptible strains, from an endemic region characterized by high strain variability and population diversity.

In addition to the strain type, fitness may be also be affected by the drug resistant mutations (and the fitness cost associated with it) and the assay system (the culture media or the host) which influences selection. In this study, using the Genotype MTBDR plus line probe assay, it was identified that the most commonly associated mutations in the drug resistant strains, in the study population (Refer Materials and Methods) were D516V (*rpoB*), S315T1/T2 (*katG*) and A16G (*inhA*). The frequency of their occurrence was 42.1%, 74.1% and 76.5% respectively (unpublished data). Studies from North India evaluating drug resistance mutation and MIC for *rpoB* have documented a low level resistance associated with D516V (1–10 µg/ml) [19] implying that this mutation by itself may not exact high fitness costs. Hence the observations in this study can be imputed to factors other than the mutations.

The assay system (which has earlier been shown to alter the measure of fitness of *M.tb* strains [17]) in this study was of non-selective nutrient media (MB7H11), which is expected to demonstrate the basic “wild-type” fitness of *M.tb* strains. Therefore, H37Rv, a standard laboratory drug susceptible strain (not exposed to selection through host and drug pressures), should demonstrate the highest fitness. This was confirmed in the observation of reduced fitness of all drug resistant strains in the presence of H37Rv.

The decreased fitness of C-MDR (0–0.26) in the presence of drug susceptible strains from the CAS lineage could be due to the fact that CAS in itself is found to be a slow growing strain with a generation time of (121.2±11.3 h) (Table 3) which is significantly higher than the drug resistant strains belonging to the Beijing and MANU lineages (p<0.005, t test). It has been observed [20] that the slow growing M.tb strains accumulate a single base pair substitution in *rpoB* locus at the rate of 10^−10^. This could imply that for a compensatory mutation to have established itself and return the strain to its original fitness would take much longer. Thus, it is likely that the mutation has associated a cost to fitness which is as yet inadequately compensated.

In contrast, the enhanced fitness of Beijing drug resistant strain (1.00–1.92) over drug susceptible strains of the CAS and Beijing lineages could be due to specific compensatory mechanisms present in the Beijing strains from this region which are significantly associated with MDR [11], [15]. It could also be due to the fact that Beijing genotype is known to be associated with several outbreaks across the world implying mechanisms which permit its predominance even in the presence of other competing strains. This highlights the virulent nature of the strain and its high transmission efficiency across different continents and thus its inherent ability to multiply faster in the presence of several strain types. However in a TB endemic region like India, native strains are strongly adapted to the population and thus may not allow Beijing strains to multiply to an epidemic proportion. We also project that given the ‘un-restricted’ nature of Beijing MDR strains, a disproportionate increase of these, in an endemic setting like ours could lead to an impending epidemic outbreak. Thus surveillance systems for strain make up in vulnerable regions may be required for timely warning of drug resistant epidemics

The MANU drug resistant strains show a high fitness index (1.44–5.51) in the presence of CAS and Beijing drug susceptible strains but do not grow in the presence of MANU drug susceptible strains. A sympatry between MANU and the host may be responsible for the predominance [14] of the strain in the region and its high fitness over the drug resistant strains of its own and other genotypes. The mutual inhibition of MANU drug resistant and susceptible strains could be indicative of an interesting phenomenon of cell-cell communication used by bacteria and mediated through diffusible signal molecules to confine the population density and to modulate their behavior in response to the environment. An analogy was found in ‘deadly’ sibling colonies of *Paenebacillus dendritiformis* mutually inhibiting each other [21].

While the MANU drug susceptible strains and H37Rv grew faster in competition (when compared to their independent growth rate), the difference was greater in H37Rv as compared to the MANU drug susceptible strains. Although this was not statistically significant, it still indicates the fitness of the H37Rv (a laboratory strain) in non-selective media, as compared to MANU (a drug susceptible clinical strain) which is probably better adapted to the selective host conditions.

Observations elsewhere indicate that even in the absence of drug pressure, drug resistant strains with relatively low basic reproductive number may nonetheless persist within co-infected hosts providing opportunities for selection, but do not out compete its relative successful competitor, the drug susceptible strains [22]. The findings from the present study are probably representative of this scenario and also highlight the possibility that the slow growing drug resistant strains may not be completely inhibited, awaiting the opportunity of resuming more rapid growth and changing the dynamics of competition.

Whilst the results demonstrate the association between non-synonymous substitutions and strain fitness they also highlight that it is the competing strain and its genotype that has a significant bearing on the fitness of the strain rather than the mutation itself. To explain this we choose the example of the MANU drug resistant strain (M-MDR) which bears non synonymous substitutions in both *rpoB* and *inhA*. If it is assumed that the substitutions bore a fitness cost which was later compensated in the bacterium it should have remained so under most *in vitro* conditions. However it was seen that though MANU strains exhibited the highest fitness index in the presence of drug susceptible CAS and Beijing strains but they were strongly inhibited by MANU drug susceptible strains.

It is most likely that the pathogen uses mechanisms other than mutations to overcome the effects of drugs, such as differential expression of genes [23], the production of bacteriocins [24] or activation of drug efflux pumps. The cost of maintenance thereof, is for the efflux pumps rather than for the mutation (which alters the target site generating resistance) and the compensatory mutation (which returns the original fitness).

We conclude that MANU drug susceptible strains are the fittest while competing with the drug resistant isolates from the 3 predominant lineages of our cohort. Consequently, it may be hypothesized, that being more fit; the predominant drug susceptible strain in an endemic locale (like MANU in Mumbai) may outgrow other strains in the region. This hypothesis bears further testing.

Overall our findings strengthen the contention that in a given geographical location, the fitness of a strain is influenced not only by a particular non synonymous substitution but also by the competing strains in the environment. The higher fitness index in such competitive environment may also help predict the predominance of a particular lineage in a given setting. Further studies are needed to understand the mechanisms that drive the interplay of inter and intra strain competition and also newer methods to determine competition between drug susceptible strains. Such methods may help elucidate the interplay of ‘nature’ and ‘nurture’ in prokaryotes.
